# Echocardiographic Assessment After Heart Transplant

**DOI:** 10.3390/diagnostics16182970

**Published:** 2026-09-14

**Authors:** Cecília Beatriz Bittencourt Viana Cruz, Juliana Barbosa Sobral-Alves, Stephan Milhorini Pio, Juliana Bittencourt Cruz Salviano, Marco Stephan Lofrano-Alves, Daniela do Carmo Rassi, Ludhmila Abrahão Hajjar, Marcelo Luiz Campos Vieira

**Affiliations:** 1 Heart Institute (InCor), Medical School, University of São Paulo, São Paulo 05403-900, Brazil; julianabsobral@gmail.com (J.B.S.-A.); stephanpio@yahoo.com.br (S.M.P.); jubittencourt180@gmail.com (J.B.C.S.); ludhmila@usp.br (L.A.H.); mluiz766@terra.com.br (M.L.C.V.); 2 Post Graduate Program in Internal Medicine, Internal Medicine Department, Medical School, Federal University of Paraná, Curitiba 80060-240, Brazil; mslalves@hotmail.com; 3 Department of Internal Medicine, Medical School, Federal University of Goiás, Goiânia 74605-040, Brazil; dani.rassi@hotmail.com

**Keywords:** heart transplantation, echocardiography, allograft rejection, cardiac allograft vasculopathy, speckle-tracking echocardiography, myocardial work, three-dimensional echocardiography, right ventricular function

## Abstract

Heart transplantation remains the gold-standard therapy for selected patients with end-stage heart failure, yet acute rejection and cardiac allograft vasculopathy (CAV) continue to limit long-term survival. While endomyocardial biopsy and coronary angiography remain the reference standards for diagnosing rejection and CAV, respectively, echocardiography offers a non-invasive, repeatable tool for the longitudinal surveillance of heart transplant recipients. This review summarizes the expected echocardiographic findings following orthotopic heart transplantation according to surgical technique (biatrial versus bicaval) and describes the echocardiographic features of early graft dysfunction, acute allograft rejection, and late graft failure due to cardiac allograft vasculopathy. We also discuss the incremental value of newer echocardiographic techniques—including speckle-tracking-derived strain, myocardial work, and three-dimensional echocardiography—in detecting subclinical left ventricular and right ventricular dysfunction that may precede changes detectable by conventional parameters. No single echocardiographic parameter reliably diagnoses rejection or vasculopathy in isolation; an integrated, multiparametric approach combined with clinical data is required. Emerging techniques hold promise for reducing reliance on invasive surveillance, although further validation in larger, multicenter cohorts is needed. This review aims to provide echocardiographers and transplant cardiologists with a practical, imaging-based framework for the follow-up of heart transplant recipients across the early postoperative period and long-term surveillance.

## 1. Introduction

Heart transplantation is the gold standard treatment for selected patients with end-stage heart failure, with significant improvement on survival and quality of life [1,2]. Although remarkable advances on surgical techniques, donor and recipient selection criteria, advances in immunosuppressive therapy, and management of transplant patients were observed in recent decades, acute rejection and cardiac allograft vasculopathy remain as important limiting factors of better survival. Even though follow-up of these two conditions is historically performed with invasive procedures such as myocardial biopsy (MB) and coronary angiography, which present inherent risks and elevated costs, echocardiography is an important tool for the follow-up of these patients [3].

Prior reviews have summarized the role of echocardiography in heart transplant recipients, including conventional assessment, rejection, and cardiac allograft vasculopathy [4]. The present review complements this literature by adopting a longitudinal, stage-based framework spanning the immediate postoperative period through late graft failure, incorporating transplant-specific diastolic assessment, contemporary evidence on left- and right-ventricular strain, myocardial work, three-dimensional echocardiography, and recent multimodality developments such as CCTA/CT myocardial perfusion, together with dedicated practical comparison tables.

Despite limitations for optimal acquisition of transthoracic echocardiographic images, shortly after cardiac transplant surgery, follow-up with serial echocardiographic studies is feasible in the majority of patients and has a fundamental role in assessing left ventricular function and diagnosing complications related to cardiac transplant. Transoesophageal echocardiography is an alternative with high accuracy in patients with inconclusive transthoracic scans. On late follow-up, echocardiography is especially useful to detect acute rejection and cardiac allograft vasculopathy and to monitor pulmonary artery pressures. Due to variability of the echocardiographic parameters in this population [5], a comprehensive transthoracic scan is necessary as a control and must be performed at least six months after heart transplantation. Subsequent echocardiographic studies should be interpreted in comparison with the data obtained from this 6-month study [6].

**Figure 1:**
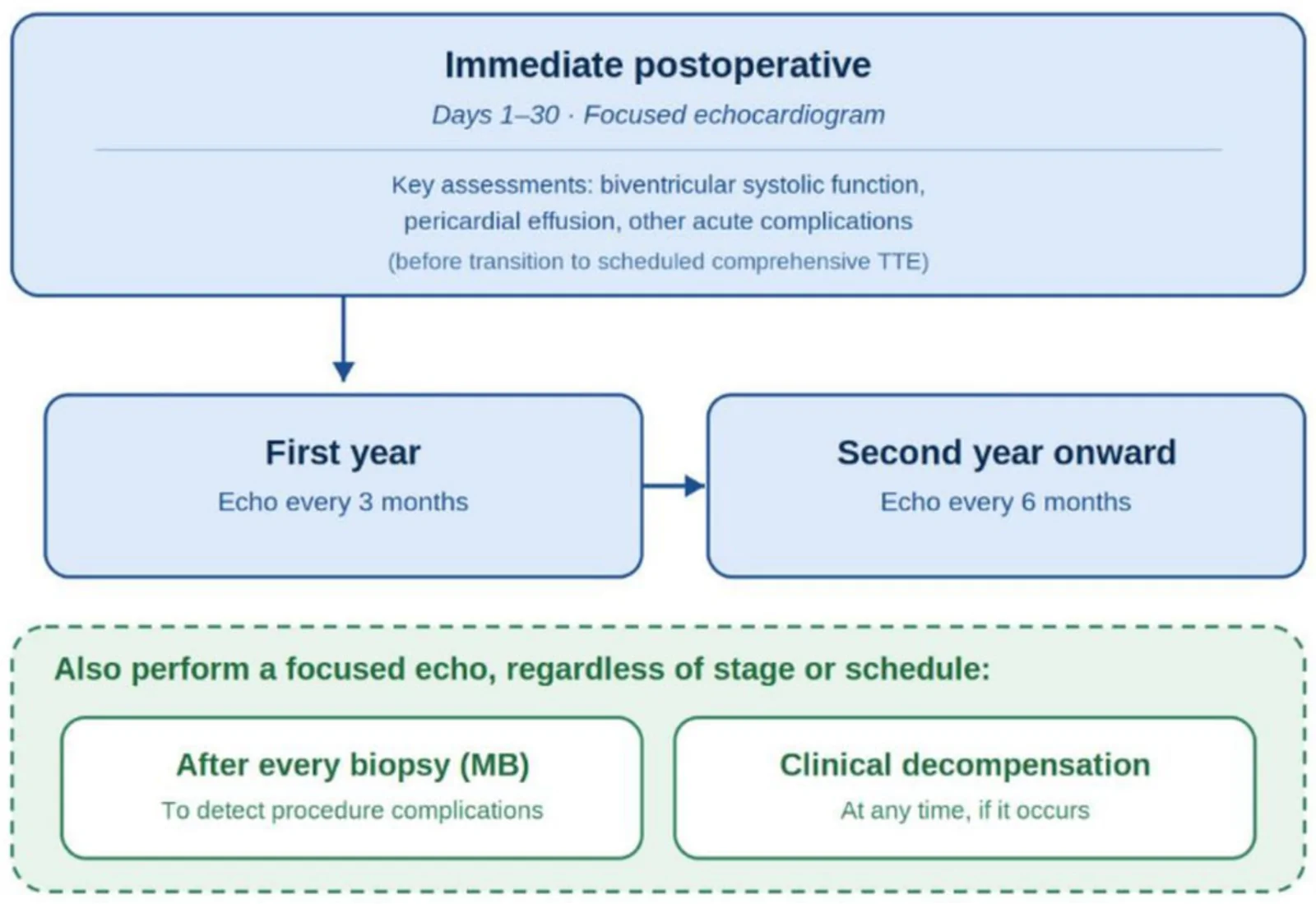
Recommended frequency of transthoracic echocardiography (TTE) during heart transplant follow-up. In addition to the scheduled intervals, a TTE should also be performed after every endomyocardial biopsy (MB) and in the presence of clinical decompensation. Abbreviations: TTE, transthoracic echocardiography; MB, myocardial biopsy.

During the first year after heart transplantation, transthoracic echocardiogram is recommended every three months, while in the second year, an echocardiogram should be done every six months. A scan also must be performed every time patients undergo MB (aiming to detect possible complications of the procedure), and in the presence of clinical decompensation [6] (Figure 1).

When an invasive diagnostic or therapeutic procedure is planned, a comprehensive echocardiographic examination performed in close temporal proximity to the procedure should also be considered whenever clinically feasible. This permits contemporaneous correlation of cardiac structure, ventricular function, hemodynamics, and valvular findings with invasive hemodynamic, angiographic, surgical, or histopathological information and provides a useful reference for subsequent longitudinal assessment. A focused echocardiographic examination after endomyocardial biopsy or another invasive procedure remains useful for detecting procedure-related complications, but it should not be considered a substitute for comprehensive graft assessment when clinically indicated.

### 1.1. Typical Echocardiographic Findings in Heart Transplant

Echocardiographic findings after cardiac transplant must be interpreted according to the aspects of surgical techniques [5]. Currently, bicaval technique is the most performed for orthotopic cardiac transplant, followed by biatrial technique (conventional) [7]. On conventional technique, donor’s heart anastomosis is performed at the medium level of recipient’s both atria and ascending aorta and pulmonary artery above semilunar valves. On the bicaval technique, anastomosis is performed at superior and inferior venae cavae instead of at the right atrium. At the left atrium, the goal is that a small portion of atrial wall around the four pulmonary veins is left. Conventional biatrial technique was associated with delayed atrial mechanics, abnormal left ventricular filling pattern, greater risk of atrial thrombi and higher incidence of tricuspid valve regurgitation. Bicaval technique, on the other hand, is associated with preservation of left atrial morphology, lower rates of pacemaker due to sinoatrial node complications, and lower thrombogenesis [8,9]. Tricuspid valve regurgitation (TR) is a common sequela immediately after heart transplantation, and its occurrence has decreased after the adoption of the bicaval anastomosis technique. Kim et al. demonstrated that in patients undergoing heart transplantation with the bicaval technique, significant TR was less common than the rates reported in previous studies and showed a trend of improvement within a year after surgery [10]. 

**Figure 2:**
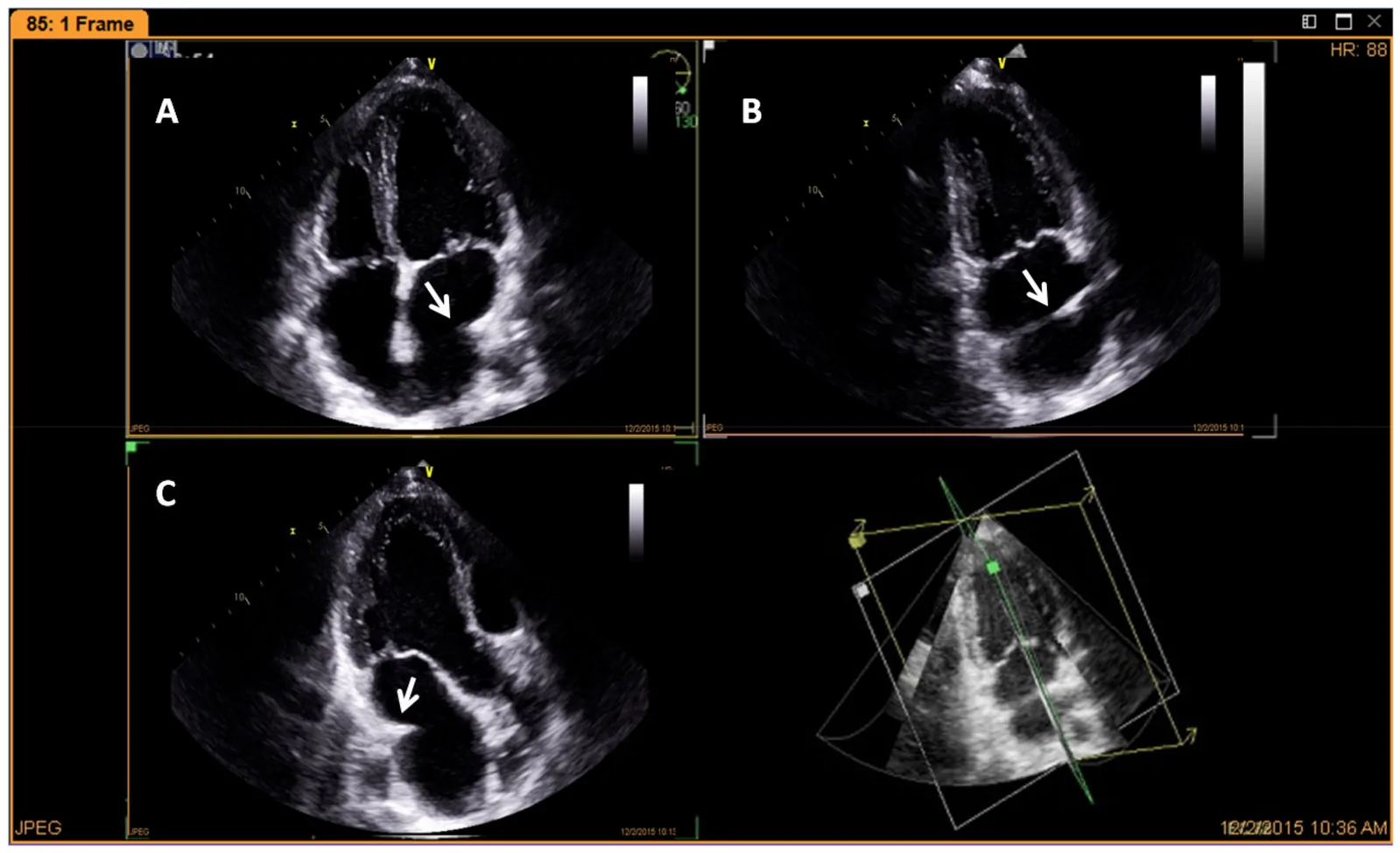
Three-dimensional echocardiographic reconstruction for anatomical correlation. Apical four-chamber (**A**), Apical two-chamber (**B**) and apical three-chamber (**C**) views showing the hyperechogenic atrial suture line (arrows) in three different vews after orthotopic heart transplantation, which should not be mistaken for an atrial mass or intracavitary thrombus.

A hyperechogenic image corresponding to atrial suture between donor’s and recipient’s atrial can be seen on echocardiography. This image is observed on both atria in the biatrial technique, or only on the left atrium in bicaval technique. Suture line is usually visible as a hyper refringent line and should not be mistaken as atrial mass or intracavitary thrombus (Figure 2).

Sutures of venae cavae, pulmonary artery and ascending aorta are usually not visible on transthoracic echocardiography in adults, but are easily identified in children.

Left atrial enlargement on the bicaval technique and biatrial enlargement on the biatrial technique are noted, mainly on their longitudinal axes. It is important to highlight that the left atrial volume has an inverse correlation with the survival of heart transplant recipients [11].

On the early days after the surgery right ventricular dilatation and systolic dysfunction, usually transient, is often observed and due to high pulmonary vascular resistance present in some heart recipients. Lately, the right chambers adapt to the new hemodynamic status [12,13]. Although right ventricular systolic function improves in most of the patients during the first week after heart transplantation, persistent dilatation and dysfunction are known markers of intrahospital mortality, with right ventricular systolic dysfunction accounting for about 20% of death early after transplant surgery [14,15].

**Figure 3:**
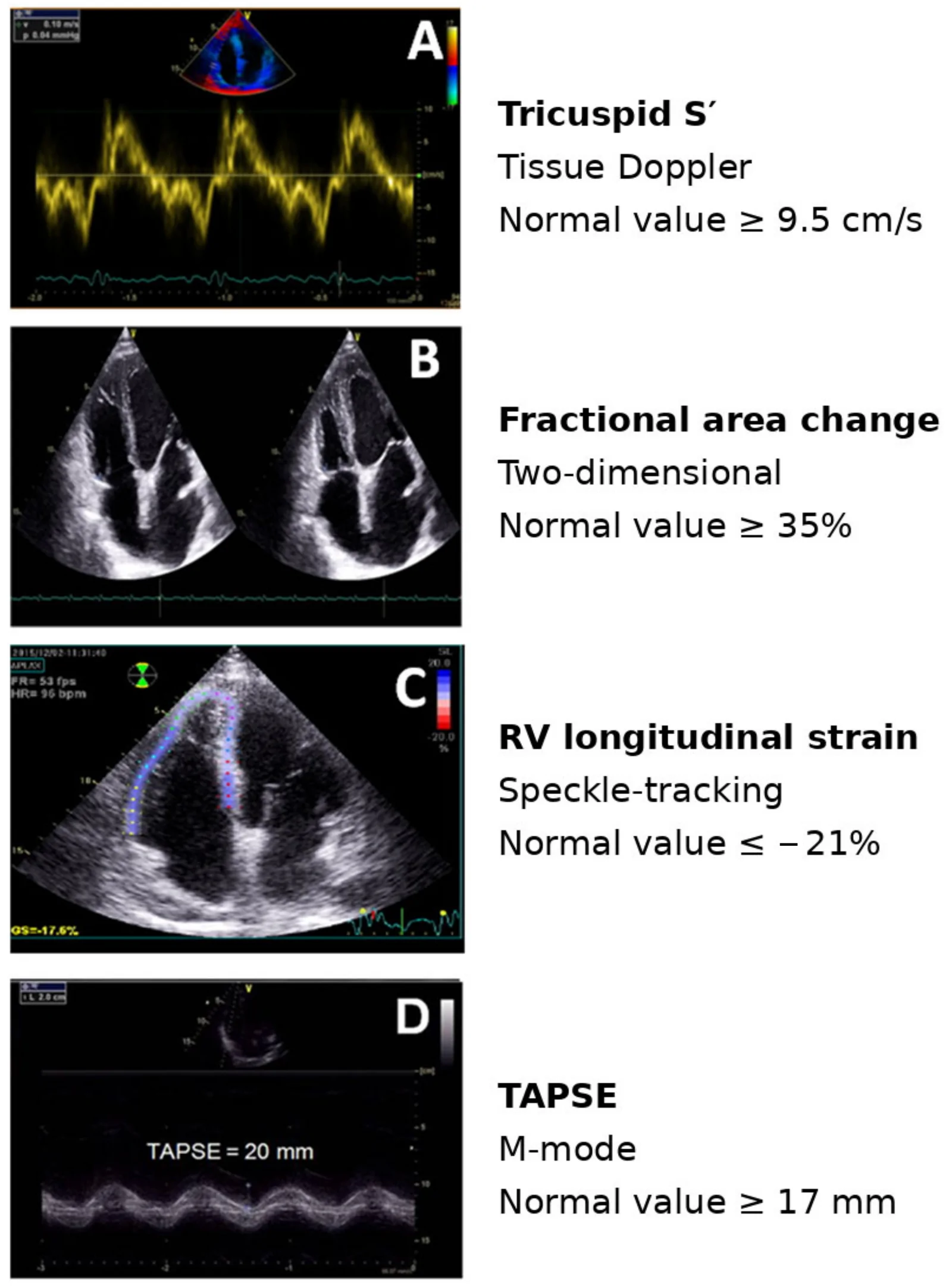
Quantitative echocardiographic parameters used to assess right ventricular systolic function after heart transplantation. (**A**) Tricuspid S′ by tissue Doppler (normal value ≥ 9.5 cm/s); (**B**) two-dimensional fractional area change (normal value ≥ 35%); (**C**) right ventricular longitudinal strain by speckle-tracking echocardiography (normal value ≤ −21%); (**D**) tricuspid annular plane systolic excursion (TAPSE) by M-mode (normal value ≥ 17 mm). Abbreviations: S′, tricuspid annular peak systolic velocity; TAPSE, tricuspid annular plane systolic excursion.

Quantitative echocardiographic parameters of the right ventricular function may uncover subtle contractility abnormalities (Figure 3) [16]. 

A recent study showed that all quantitative parameters of right ventricular systolic function in cardiac transplanted patients were reduced compared to guideline recommendations for non-transplant patients [17,18]. Nevertheless, it is recommended, whenever possible, to include these parameters in the report for comparison purposes with the patient’s own baseline values. The main echocardiographic techniques used in heart transplant recipients, together with their principal advantages, limitations, and potential clinical applications, are summarized in Table 1.

Asynchronous movement of ventricular septum and pericardial effusion are common findings after surgery. Another usual finding is thickening of the septum and posterior wall caused by postoperative edema which tends to reduce over time [6]. These and other transplant-specific echocardiographic characteristics should be considered when interpreting findings in clinically stable heart transplant recipients (Table 2).The main echocardiographic techniques used in heart transplant recipients, together with their advantages, limitations, and potential clinical applications, are summarized in Table 1.

### 1.2. Early Graft Dysfunction Following Heart Transplantation

Early graft dysfunction failure consists of systolic dysfunction of both ventricles associated with low cardiac output and elevated filling pressures and is the main cause of death in the first 30 days after heart transplantation. It is a consequence of pulmonary hypertension and right ventricular failure, reperfusion injury (prolonged cold ischemia time) or hyperacute rejection (due to the presence in the recipient of pre-existing antibodies that react with the cardiac graft) [3,19]. Bedside echocardiogram is useful for identifying biventricular systolic dysfunction, thus corroborating for the diagnosis.

### 1.3. Acute Allograft Rejection

Acute allograft rejection usually occurs in the first months after heart transplantation, although it can also be identified later in the post-transplant course. The estimated incidence is 20% to 40%, and it is the main cause of death during the first year after surgery. It is caused by a recipient’s immune-mediated response to donor’s major histocompatibility complex antigens, which can be mediated by cellular reaction (acute cellular rejection) or by antibodies (acute humoral rejection). Being initially asymptomatic, it requires systematic surveillance mainly during the first six months after transplant, with close monitoring of immunosuppression and clinical and laboratory data. Even though endomyocardial biopsy remains the gold standard for rejection diagnosis, it has several limitations, including sampling error, interobserver variability in the interpretation of histological findings, the frequency required for surveillance, procedure-related complications, and the fact that it samples only the right ventricle [20].

Two-dimensional echocardiography with color Doppler is a useful tool for evaluating changes in allograft’s structure and function which are related to acute cellular rejection. Nevertheless, upon initial or mild acute cellular rejection, changes in echocardiographic parameters are subtle and non-specific or reproducible enough to allow for adjustment on immunosuppression drugs [21].

The first described echocardiographic sign related to rejection was an increase in the thickening of the ventricular walls resulting from interstitial edema. Changes in ventricular thickness, however, can be subtle in most of the cases, with low sensitivity and specificity [22]. Occurrence of pericardial effusion is common after cardiac transplant, although worsening of effusion was related to acute rejection [23,24].

Heart transplantation introduces unique physiological changes that complicate the assessment of left ventricular (LV) diastolic function. Cardiac denervation results in persistent sinus tachycardia, frequently causing fusion of mitral E and A waves and limiting the interpretation of conventional Doppler parameters. Furthermore, the transplanted heart undergoes dynamic changes during the early postoperative period, including myocardial edema, altered loading conditions, and gradual recovery of myocardial tissue velocities.

In patients with preserved ejection fraction (EF), predominant early diastolic filling (E/A ≥ 2) is a common physiological finding because donor hearts are typically obtained from young, healthy individuals. Therefore, a restrictive-looking mitral inflow pattern should not be interpreted as evidence of elevated filling pressures in isolation. Likewise, pulmonary venous flow is of limited value because the S/D ratio is frequently reduced in transplant recipients.

Although LV diastolic dysfunction may occur during acute graft rejection due to myocardial edema or during chronic rejection because of myocardial fibrosis, no single echocardiographic diastolic parameter reliably identifies rejection. Instead, assessment should focus on estimating LV filling pressures using an integrated approach.

Importantly, no universally validated magnitude of change in wall thickness, Doppler indices, tissue velocities, ventricular function, or deformation parameters can currently be considered diagnostic of rejection in an individual patient. Serial measurements are affected by technical test–retest variability, image quality, loading conditions, heart rate, analytical method, and inter- and intra-observer variability. Small changes may therefore fall within expected measurement or biological variation. Echocardiographic abnormalities should be considered supportive or suggestive—particularly when changes are substantial, reproducible, and concordant across several parameters—and should prompt integration with clinical findings, biomarkers, invasive hemodynamics, and endomyocardial biopsy rather than independently determine a diagnosis of rejection.

**Figure 4:**
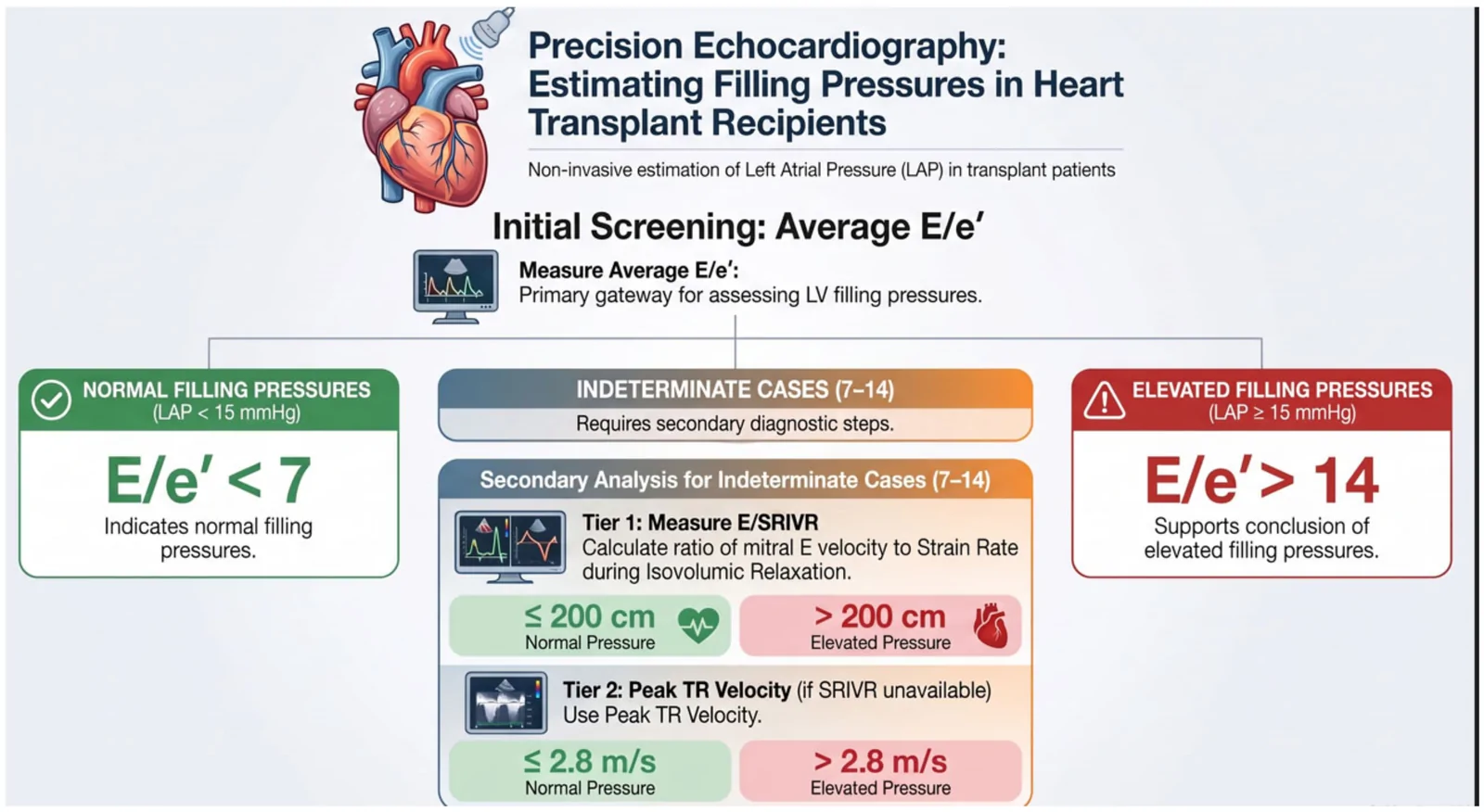
Algorithm for the Assessment of Diastolic Function in Heart Transplant Recipients. Abbreviations: LV, Left Ventricular; E, peak early diastolic mitral inflow velocity; e′, peak early diastolic mitral annular velocity (average of septal and lateral); SRIVR, strain rate during the isovolumic relaxation period; TR, tricuspid regurgitation; LAP, left atrial pressure.

A simplified algorithm has been validated for heart transplant recipients in sinus rhythm: [25] (Figure 4)

• Average E/e′ < 7: normal LV filling pressures.
• Average E/e′ > 14: elevated LV filling pressures.
• Average E/e′ 7–14: measure LV strain rate during isovolumic relaxation (SRIVR) from all three apical views.
  ∘ Mitral E/SRIVR ≤ 200 cm: normal LV filling pressures.
  ∘ Mitral E/SRIVR > 200 cm: elevated LV filling pressures.
• If SRIVR is unavailable: use peak tricuspid regurgitation (TR) velocity.
  ∘ Peak TR velocity ≤ 2.8 m/s: normal LV filling pressures.
  ∘ Peak TR velocity > 2.8 m/s: elevated LV filling pressures.

Mitral annular e′ velocities should be interpreted cautiously because they are influenced by translational cardiac motion and are typically reduced in the early postoperative period before gradually increasing over subsequent weeks and months.

### 1.4. Late Graft Failure Due to Cardiac Allograft Vasculopathy

Cardiac allograft vasculopathy is characterized by a fibroproliferative disorder (diffuse fibrous intimal hyperplasia and proliferation of smooth muscle cells) as result of cumulative endothelial injury, leading to concentric narrowing and occlusion of coronary arteries. Its prevalence at 3, 5 and 10 years after heart transplantation is, respectively, 20%, 30% and around 50% [3]. Clinical diagnosis is challenging because of the absence of typical symptoms of angina pectoris in transplanted patients as a consequence of sympathetic afferent denervation. Heart failure, ventricular arrhythmia and sudden cardiac death are often the early clinical manifestations of this condition. Treatment comprises proliferation signal inhibitors, antiplatelet drugs, statins and coronary revascularization whenever possible [26].

Coronary angiography is the gold standard for diagnosis. This method, however, can underestimate the extent and severity of this condition due to its diffuse nature and involvement of distal vessels, with increase in sensitivity with associated use of intravascular ultrasound (IVUS). Furthermore, because angiography is an invasive procedure with elevated costs, most centers perform coronary angiography with IVUS at regular intervals after the first year of transplant, alternating with non-invasive stress methods [27,28].

Non-invasive imaging of CAV has advanced considerably in recent years. Coronary computed tomography angiography (CCTA) allows direct visualization of epicardial vessel wall thickening and has been incorporated as a class IIa recommendation for CAV surveillance in the 2023 International Society for Heart and Lung Transplantation (ISHLT) guidelines, reflecting growing confidence in its diagnostic performance relative to invasive angiography [29]. Dynamic computed tomography myocardial perfusion imaging, combined with CCTA, has further improved the detection of both epicardial and microvascular components of CAV, addressing a key limitation of angiography alone [30]. Cardiac magnetic resonance imaging, through T1/T2 mapping and late gadolinium enhancement, offers complementary information on myocardial edema and fibrosis associated with chronic allograft injury, although its role in routine CAV surveillance is still being defined [29].

Rest echocardiography has low sensitivity to detect cardiac allograft vasculopathy. Some studies, however, showed high specificity, and presence of wall motion abnormalities at rest should trigger investigation of cardiac allograft vasculopathy by invasive method [31]. Stress echocardiography is a surveillance tool for this condition [28]. Nonetheless, sensitivity and predictive value of this method rely on enough hemodynamic stress to reach ischemic threshold, often difficult to accomplish in transplant patients due to chronotropic incompetence in many cases [32].

Dobutamine stress echocardiography (DSE) appears as a good tool for this population. Its sensitivity in diagnosing coronary obstruction greater than 50% ranges from 50% to 100% when compared to gold standard angiography [33]. Interestingly, DSE low specificity (around 50–60%) increases when the gold standard is coronary angiography plus IVUS, which suggests that part of the “false positive” on DSE in this population is correctly diagnosed vasculopathy by wall motion abnormalities though underestimated by conventional coronary angiography. Furthermore, it suggests that DSE is capable of diagnosing coronary microvascular disease, which is undetected by arteriography [27].

A large proportion of transplant patients, however, do not reach an adequate heart rate with dobutamine as the stress agent. It is important to highlight that the use of atropine as vagolytic agent in association with dobutamine is not well validated in this population due to the section of vagus nerve after orthotopic heart transplant and the absence of vagal reinnervation in the long term [34,35]. Echocardiography with vasodilation using dipyridamole can be used as an alternative in cases of poor chronotropic response [36]. However, studies using this method are scarce, performed at few transplant centers and with limited number of patients, therefore, needing validation.

The main reason for using pharmacological stress echocardiography in transplant patients is its prognostic value. Absence of wall motion abnormalities in a submaximal scan is related to the absence of cardiac allograft vasculopathy and better prognosis in different studies. Cardiac allograft vasculopathy-related wall motion abnormalities identify patients with increased risk of cardiac adverse events. DSE sensitivity, however, varies according to follow-up duration, decreasing to about 60% when the mean follow-up was 3 years after index scan [27,35]. Therefore, for adequate prognostic value, stress echocardiography must be performed at regular intervals of one year, approximately.

### 1.5. New Echocardiographic Techniques

Over the last years new echocardiographic techniques such as speckle-tracking echocardiography (STE), automatic digital border definition methods and three-dimensional (3D) echocardiography have been studied in order to increase echocardiographic accuracy in heart transplant patients.

### 1.6. Speckle-Tracking Echocardiography

Speckle-tracking echocardiography is progressively employed to assess strain following HTx and may assist in the disclosure of rejection and CAV [37,38].

From a technical perspective, STE quantifies myocardial deformation by tracking acoustic speckles frame-by-frame throughout the cardiac cycle. In transplant recipients, longitudinal deformation is particularly relevant because subtle graft dysfunction may occur despite a preserved left ventricular ejection fraction. Acquisition and analysis should therefore be standardized: apical views should avoid foreshortening, frame rate and image quality should be optimized, the region of interest should follow the myocardial contour, and serial examinations should preferably use the same acquisition protocol and analysis software. This is especially important in longitudinal surveillance because absolute strain values are influenced by loading conditions, image quality, analysis methodology, and software. Although contemporary platforms show substantially improved agreement, residual inter-vendor variability remains; consequently, changes from an individual post-transplant baseline may be more informative than isolated thresholds [18,39].

In heart transplant patients with cardiac allograft cardiopathy, STE-derived left ventricular global longitudinal strain was lower when compared to healthy individuals, and correlated with the severity of this condition [40]. In addition, strain rate obtained from DSE showed good accuracy in the diagnosis of cardiac allograft vasculopathy [41].

In patients with grade ≥ 2R rejection, left ventricular global longitudinal strain and right ventricular free wall strain were reduced, and both parameters when combined could identify patients with small chances of rejection [42]. Left ventricular mechanics abnormalities in acute cellular rejection were assessed by ventricular twist derived from STE. When rejection is considered, a 25% reduction of left ventricular twist compared with basal values was a predictor of grade ≥ 2R acute cellular rejection with a positive predictive value of 92.9% [43]. Besides that, the lack of left ventricular global longitudinal strain improvement after cardiac transplant was associated with death and cardiac events [44].

The presence of abnormal longitudinal strain with a compensatory increase in circumferential strain (CS) parameters was seen in the early post-HTx period. These changes in echocardiography were normalized by 1 year post-transplant and remained the same over time in the absence of graft complications [45].

Tseng et al. demonstrated the use of two-dimensional speckle-tracking echocardiography (2D-STE) in severe rejection in heart transplant recipients with preserved LVEF. They observed significantly elevated early diastolic longitudinal strain rate (*p* = 0.02) and decreased global circumferential strain (GCS; *p* < 0.001) and GCS rate (*p* = 0.02) for the rejection group compared with the control group. The sensitivity and specificity of GCS to detect severe acute rejection were observed as 81.8 and 68.4%, respectively [38].

In patients with acute cellular rejection (ACR) grade ≥ 2R, the association of strain parameters derived from 2D-STE, particularly right ventricular free wall longitudinal strain, with troponin seems to be able to detect acute cellular rejection with good accuracy [46].

A recent systematic review and meta-analysis pooling data from more than 2000 paired biopsies across 18 studies reported differences in left- and right-ventricular strain between patients with and without acute cellular rejection and greater longitudinal change in LV GLS during rejection episodes [47]. These findings support further evaluation of STE as a complementary surveillance tool; however, the reported group-level differences do not establish a universally applicable cutoff or a minimum change that reliably diagnoses rejection in an individual patient. Substantial overlap between distributions, differences in acquisition and analysis, loading conditions, and intrinsic test–retest variability must be considered. In clinically stable outpatients, RV strain may identify subtle abnormalities not apparent on conventional parameters [48], but such findings should be interpreted relative to a technically comparable individual baseline and in conjunction with clinical, biological, and invasive information rather than as stand-alone evidence of rejection.

Myocardial work (MW) has emerged as an investigational approach to myocardial function assessment. It combines GLS with an estimated non-invasive LV pressure curve in an attempt to account for afterload and the relationship between myocardial deformation and loading conditions [49]. Although this framework may provide information complementary to LVEF and GLS, MW incorporates several measured and estimated components, each of which contributes to test-to-test variability. Consequently, evidence of group-level differences should not be extrapolated to individual diagnostic thresholds, and the clinical utility of MW in routine post-transplant surveillance remains to be established.

In a recent prospective study, Otto et al. studied echocardiographic markers associated with acute cellular rejection in a group of Chagas disease heart transplant patients. MW analysis observed increased global work efficiency in positive acute cellular rejection [50].

Beyond rejection surveillance, non-invasive myocardial work has also been explored as a marker of cardiac allograft vasculopathy, with differences in global work efficiency and global constructive work reported in transplant recipients with extensive coronary disease [51]. These findings are hypothesis-generating rather than sufficient for individual clinical decision-making. Because myocardial work is derived from strain together with an estimated non-invasive LV pressure curve, variability in image acquisition, strain tracking, blood-pressure measurement, timing, and loading conditions may propagate into the final indices. Its real-world incremental value and clinically meaningful longitudinal thresholds in heart transplant recipients therefore remain uncertain, and MW should currently be regarded as an emerging complementary research tool rather than a stand-alone diagnostic marker of rejection or CAV.

### 1.7. Three-Dimensional Echocardiography

Three-dimensional echocardiography offers technical advantages for anatomical definition and for quantification of ventricular and atrial volumes, and it is relatively accessible in centers with appropriate equipment and expertise, particularly for geometric assessment of the right ventricle, without ionizing radiation [6,52]. Studies have explored 3D-derived mechanical dyssynchrony and stress 3D echocardiography in acute rejection and cardiac allograft vasculopathy; however, these data are derived from limited research cohorts, and there is insufficient evidence to support established routine clinical utility or widespread adoption for rejection or CAV surveillance across heart-transplant programs. Therefore, the current clinical value of 3D echocardiography in this setting is primarily related to improved chamber quantification, whereas its role in diagnosing rejection or CAV remains investigational and requires prospective multicenter validation to determine whether it provides clinically meaningful incremental value beyond conventional echocardiography and established multimodality surveillance.

Relationship to previous reviews: Masarone et al. provided a clinically oriented overview of echocardiography in heart transplant recipients, including conventional assessment, rejection, cardiac allograft vasculopathy, and deformation imaging [4]. The present review complements that work by emphasizing a longitudinal, stage-based imaging framework from the immediate postoperative period through late graft failure, incorporating transplant-specific diastolic assessment, contemporary evidence on left- and right-ventricular strain, myocardial work, three-dimensional echocardiography, and recent multimodality developments such as CCTA/CT myocardial perfusion. In addition, the current review highlights practical differences between the transplanted and non-transplanted heart and summarizes the strengths and limitations of available echocardiographic techniques in dedicated tables.

### 1.8. Discussion and Future Directions

Echocardiography has an important but intrinsically complementary role in the surveillance of heart transplant recipients. Conventional parameters have limited sensitivity and specificity for early or mild rejection and are influenced by graft-specific physiology, loading conditions, image acquisition, and measurement variability. Advanced techniques—including speckle-tracking-derived strain, myocardial work, and three-dimensional echocardiography—have demonstrated associations with graft abnormalities in research cohorts, but statistically significant group-level differences should not be interpreted as validated diagnostic thresholds for individual patients. Substantial overlap between groups and intrinsic test–retest variability may limit the clinical meaning of small changes on serial examinations. Accordingly, echocardiographic findings should be interpreted together with clinical status, biomarkers, histopathology, invasive hemodynamics, coronary assessment, and other imaging modalities. Serial comparison with a technically comparable individual baseline is preferable whenever possible, while endomyocardial biopsy and established coronary reference methods remain necessary when clinically indicated [6,47].

Future research should prioritize prospective multicenter protocols using standardized acquisition, core-laboratory analysis, and predefined longitudinal endpoints. A major unmet need is the determination of test–retest reproducibility and reference change values for each echocardiographic parameter in clinically stable transplant recipients. Rather than extrapolating statistically significant differences between groups to individual patients, studies should establish the magnitude of longitudinal change that exceeds expected technical and biological variability and is meaningfully associated with rejection, graft dysfunction, or CAV. Integration of echocardiographic findings with biomarkers, donor-derived cell-free DNA, histopathology, invasive hemodynamics, coronary assessment, and cross-sectional imaging may enable more reliable risk-adapted surveillance pathways. Automated and AI-assisted quantification could improve reproducibility, but these tools require prospective transplant-specific validation before they can influence decisions to defer biopsy or invasive coronary assessment.

For cardiac allograft vasculopathy specifically, the growing acceptance of coronary computed tomography angiography in contemporary guidance signals a broader shift toward multimodality graft surveillance, in which echocardiographic findings should be integrated with—rather than substituted for—invasive, biological, clinical, and cross-sectional imaging information [29,30]. Artificial-intelligence-assisted analysis may eventually help reduce measurement variability through automated chamber and deformation quantification [53]. A particularly promising research direction would be prospective multimodal AI models trained against clinically meaningful reference outcomes, incorporating echocardiographic, histopathological, invasive hemodynamic, biomarker, and clinical data. Multicenter or federated-learning networks involving heart-transplant programs could broaden population heterogeneity while allowing institutions to retain local data, potentially reducing institution-specific overfitting and improving external validity. Such systems would require standardized acquisition, rigorous human supervision, prospective external validation, transparent performance monitoring, and safeguards against erroneous or non-generalizable outputs before clinical implementation. Until robust transplant-specific evidence is available, echocardiographic parameters should remain complementary to established diagnostic standards.

## 2. Conclusions

The contraction pattern of the transplanted heart has specific characteristics, and conventional echocardiographic parameters and strain values may differ from those observed in the general population. Echocardiography provides non-invasive structural, functional, and hemodynamic information throughout follow-up, but its findings should not be interpreted as stand-alone diagnostic criteria for rejection or cardiac allograft vasculopathy. Endomyocardial biopsy remains the reference standard for rejection, and multimodality assessment remains essential. Speckle-tracking-derived strain and other advanced parameters may identify subclinical abnormalities and contribute to risk stratification, particularly when substantial changes are observed relative to a technically comparable individual baseline. However, test–retest variability, loading conditions, acquisition and analysis differences, and the absence of validated transplant-specific thresholds limit individual-level interpretation. Accordingly, echocardiographic findings should be integrated with clinical, biological, histological, hemodynamic, and other imaging data, and further prospective studies are needed to define reproducibility and clinically meaningful longitudinal changes before advanced parameters can be used to guide invasive surveillance decisions.

## Figures and Tables

**Table 1:** Main advantages, limitations, and potential applications of echocardiographic techniques in heart transplant recipients.

Technique	Main Advantages	Main Limitations	Potential Role After HTx
Conventional 2D TTE	Widely available; bedside; repeatable; evaluates chambers, valves, pericardium and global systolic function.	Low sensitivity for mild or subclinical rejection; measurements are affected by transplant-specific geometry and loading.	Routine surveillance; postoperative complications; serial comparison with individual baseline.
Doppler/tissue Doppler	Provides hemodynamic and diastolic information; estimates filling pressures and pulmonary pressures.	Denervation-related tachycardia may fuse E and A waves; e′ and other indices vary with postoperative recovery and loading.	Integrated assessment of filling pressures and graft hemodynamics.
Speckle-tracking echocardiography	Detects subclinical LV and RV dysfunction despite preserved EF; useful for serial assessment; potential rule-out value for rejection.	Load- and image-quality dependent; residual inter-vendor/software variability; transplant-specific cutoffs are not fully standardized.	Rejection surveillance; CAV assessment; longitudinal graft-function monitoring.
Stress echocardiography	Non-invasive functional assessment; provides prognostic information; may reveal inducible wall-motion abnormalities.	Chronotropic incompetence may limit exercise or dobutamine stress; variable sensitivity for diffuse CAV.	Non-invasive surveillance for CAV, particularly when invasive testing is not immediately indicated.
Three-dimensional echocardiography	More accurate chamber volumes and RV geometry; improved anatomical assessment; avoids geometric assumptions.	Dependent on acoustic window, temporal/spatial resolution and expertise; limited transplant-specific outcome data.	Quantification of LV/RV volumes and function; anatomical assessment; potential adjunct during stress.
Myocardial work	Integrates strain with estimated LV pressure and partially accounts for afterload; may add information beyond GLS.	Requires high-quality strain and blood-pressure data; limited validation and reference ranges in HTx.	Emerging marker for rejection and CAV; research and advanced surveillance.

**Table 2:** Practical echocardiographic differences between non-transplanted individuals and clinically stable heart transplant recipients.

Parameter/Consideration	Non-Transplanted Heart	Heart Transplant Recipient	Practical Implication
Heart rate/innervation	Normal autonomic innervation and heart-rate variability.	Cardiac denervation causes higher resting heart rate and altered chronotropic response.	Interpret Doppler filling and stress response in the context of denervation.
Atrial morphology	Normal atrial anatomy.	Atrial enlargement and suture-line morphology depend on biatrial vs. bicaval surgical technique.	Do not misinterpret the atrial suture line as thrombus or mass; account for surgical technique.
LV systolic function	Reference EF and GLS values derived from general populations.	EF may remain preserved while GLS is mildly reduced even in stable recipients.	Serial change from the patient’s own baseline is important; subtle strain reduction is not diagnostic of rejection in isolation.
RV size and function	Standard guideline reference ranges generally apply.	RV size is often larger and longitudinal indices such as TAPSE and RV strain may be lower after HTx.	General-population thresholds may overcall RV dysfunction; integrate FAC, S′, strain, 3D assessment and clinical context.
Diastolic assessment	Standard algorithms generally applicable.	Tachycardia, E/A fusion, postoperative edema and altered tissue velocities complicate interpretation.	Use an integrated approach to filling pressures; avoid relying on a single diastolic parameter.
Tricuspid regurgitation	Usually reflects primary/secondary valve disease.	Common after HTx and influenced by surgical technique, RV loading and repeated biopsies.	Assess mechanism and severity longitudinally rather than interpreting TR in isolation.
Pericardial effusion/wall thickness	New changes usually prompt evaluation for conventional causes.	Small effusion and transient wall thickening can be postoperative findings; interval worsening may raise concern for rejection.	Comparison with prior studies and timing after transplantation are essential.
Longitudinal follow-up	Population reference ranges are often sufficient.	Marked inter-individual variability makes a stable post-transplant baseline particularly valuable.	Use standardized acquisition and compare serial studies with the same patient’s baseline whenever possible.

HTx, heart transplantation; LV, left ventricular; RV, right ventricular; EF, ejection fraction; GLS, global longitudinal strain; TAPSE, tricuspid annular plane systolic excursion; FAC, fractional area change. Adapted conceptually from transplant imaging recommendations and transplant-specific reference data [6,18].

## Data Availability

No new data were created or analyzed in this study. Data sharing is not applicable to this article.
